# Development of an Automatic Dispensing System for Traditional Chinese Herbs

**DOI:** 10.1155/2017/9013508

**Published:** 2017-09-07

**Authors:** Chi-Ying Lin, Ping-Jung Hsieh

**Affiliations:** Department of Mechanical Engineering, National Taiwan University of Science and Technology, Taipei City, Taiwan

## Abstract

The gathering of ingredients for decoctions of traditional Chinese herbs still relies on manual dispensation, due to the irregular shape of many items and inconsistencies in weights. In this study, we developed an automatic dispensing system for Chinese herbal decoctions with the aim of reducing manpower costs and the risk of mistakes. We employed machine vision in conjunction with a robot manipulator to facilitate the grasping of ingredients. The name and formulation of the decoction are input via a human-computer interface, and the dispensing of multiple medicine packets is performed automatically. An off-line least-squared curve fitting method was used to calculate the amount of material grasped by the claws and thereby improve system efficiency as well as the accuracy of individual dosages. Experiments on the dispensing of actual ingredients demonstrate the feasibility of the proposed system.

## 1. Introduction

Traditional Chinese medicine (TCM) can be divided into herbal decoctions and commercial extracts [1]. The former involves boiling TCM herbs to obtain a liquid for direct administration, whereas the latter involves artificial processing, such as drying, after the boiling process to produce a powder. Commercial extracts are easier to produce, transport, and administer. Their price is also more competitive. In contrast, herbal decoctions must be formulated by patients and the herbs tend to be more expensive. As a result, commercial extracts are preferred by many practitioners. Whether herbal decoctions and commercial extracts differ with regard to efficacy remains a topic of academic debate [2, 3]. Most TCM prescriptions require the boiling of multiple medicinal herbs followed by complex solubilizing, precipitation, adsorption, suspension, and chemical reactions to create new substances with enhanced efficacy, reduced potency, or reduced toxicity. Commercial extracts are mixtures of boil-free granules that have not undergone complex chemical reactions [4, 5]. The production process destroys many volatile substances and necessitates the addition of excipients (mainly starch), which reduces the concentration of the herbal extracts. Furthermore, many TCM practitioners are accustomed to prescribing herbal decoctions in accordance with the medication compliance of their patients and then adjusting the prescriptions according to their constitution and specific symptoms. As a result, herbal decoctions remain an important part of clinical TCM.

Statistics compiled by the HPSO/CNA on pharmacist negligence [6] indicate that between 2002 and 2011, 43.8% of pharmacist negligence involved dispensing the wrong drug, and 31.5% involved dispensing the wrong dosage. Furthermore, 57.9% of all deaths caused by pharmacist negligence were caused by overdose, which resulted in USD 10,454,468 in compensation for damages. The most common reasons for dispensing errors include excessive work volume (21%), insufficient pharmacy staff (12%), time restrictions (11%), overwork (11%), and interruptions during dispensing (9.4%) [7]. Dispensing errors and medical staff shortages led to the development and wide-scale implementation of automated dose dispensing (ADD) systems [8–12]. According to Pedersen et al. [13], over 53% of the hospitals in Canada and 89% of those in the US are currently using ADD systems. Studies have proven that ADD systems can lower the chance of dispensing errors [14–16], enhance overall efficiency [14, 17], and even improve space usage [18, 19]. In contrast, most TCM pharmacies store medicinal herbs in cabinets and rely on the manual retrieval and weighing of herbs, which tends to be inefficient and arduous. As a result, patients must wait longer to pick up their prescriptions and dispensing errors are common. Several years ago, HollySys developed an ADD system for the scientific concentration of herbal medicines in hospitals [20]. The ability of that system to identify drugs automatically helps to prevent dispensing mistakes; however, errors still occurred in 10% of the samples weighing more than 5 g. Song et al. [21] constructed a dispensing system for 400 types of commercial extracts. The system comprises multiple workstations and conveyor belts arranged in a parallel configuration. That system is able to fill several prescriptions at the same time and package medicines automatically.

Unfortunately, those dispensing systems are applicable only to commercial extracts. No existing scheme has been developed to automate the retrieval and dispensing of materials for herbal decoctions. This is the first study to develop such a system. The proposed solution uses a robotic arm-based ADD system in conjunction with image recognition technology. We also designed a user-friendly human-machine interface for inputting prescriptions, including the names and weights of each herb. The system automatically dispenses the medicines in the form of packets. Simulations were used to emulate the actual motions involved in retrieving herbs from cabinets in TCM pharmacies. The retrieval of herbs alters the distribution of ingredients in cabinet drawers, thereby altering the optimal retrieval location for the robotic arm. Thus, we employed machine vision to estimate the optimal location for retrieval. Unlike powdered herbs, the amount of which can be controlled using a flow control valve [21], most of the materials in herbal decoctions are irregular in size and shape. Thus, we employed a robotic claw for the retrieval of herbs. We employed the least-squares (LS) method to obtain the parameters for the retrieval equation used to calculate the weight of the herb and the size of the claw aperture. Repeated performance of retrieval trials enabled the iterative optimization of the equation. We then compared this approach with two other methods that do not use iterative refinement. Experimental results demonstrate that the proposed approach can indeed enhance the retrieval accuracy and execution efficiency of the ADD system.

## 2. Methodology

### 2.1. Prototype System

**Figure 1:**
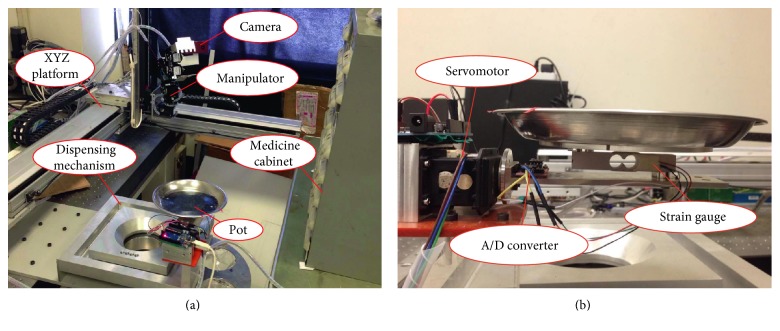
System setup. (a) Front view. (b) System used for weighing of herbs.

Figure 1(a) presents a front view of the system, which uses an XYZ linear motion platform and a 3 degrees of freedom (DOF) robotic arm, which was designed in-house. The XYZ platform is responsible for large-stroke motions using an EzM-56L-A servomotor (manufactured by FASTECH) as an actuator with an encoder resolution of 10,000/Rev. The effective operating range of the platform in the X and Y directions is 500 mm × 500 mm with a screw lead of 10 mm/Rev. The effective operating range in the Z direction is 400 mm with screw lead of 5 mm/Rev. The main tasks of the 3 DOF robotic arm are the opening of cabinet drawers and the retrieval of herbs. Servomotors manufactured by ROBOTIS were installed at the joints of the arm. MX-106 servomotors were used for the first and second axes, which resulted in resolution of 0.087°. RX-64 servomotors are used for the third axis and the claw, resulting in resolution of 0.29°. The tracking of targets using the robotic arm requires an understanding of the external environment, such as posture, location, and color; therefore, we installed a binocular stereo camera on the third axis to estimate the location of the target. The weight of the herbs was measured using a TAL220 strain gauge (manufactured by HT Sensor), which can measure up to 5 kg. We also used an HX711 24-bit A/D conversion chip to enhance measurement resolution, as shown in Figure 1(b). A computer is used to issue commands pertaining to the location of the XYZ platform and run the image processing algorithm. An Arduino board is used to acquire readings from the strain gauge and send them to the computer via an RS-232 serial communication port for subsequent analysis.

### 2.2. System Flow

**Figure 2:**
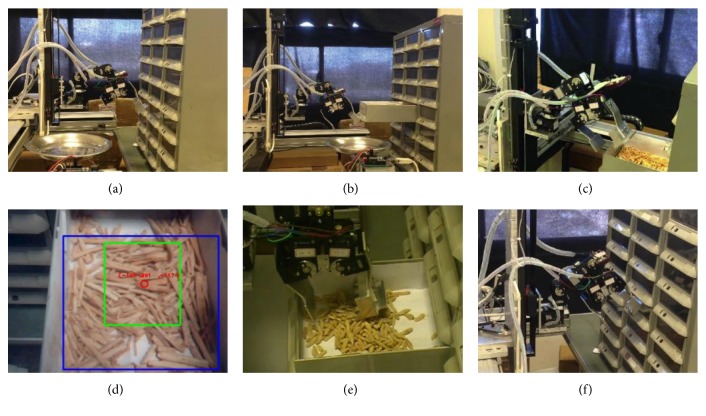
System in action. (a) Arm moving to location of herb drawer. (b) Medicine cabinet being opened. (c) Camera scanning the distribution of herbs in the drawer. (d) Determination of ideal gripping position. (e) Arm retrieving herb. (f) Medicine cabinet being closed.

Figures 2() and 3() illustrate the system in action as well as details of the various procedures. After the user inputs the names and weights of the herbs and the number of packets needed, the system accesses information pertaining to the desired herbs in a database that was created beforehand. This information includes the location of the drawer in which the herb is stored, an image of the herb, and the relationship between the weight of the herb and width of the claw used to retrieve it. The robotic arm then moves to the designated drawer and pulls it open, as shown in Figures 2(a) and 2(b). The camera scans the distribution of the herbs in the drawer and processes the image to determine the optimal retrieval location, as shown in Figures 2(c) and 2(d). The red circle in Figure 2(d) indicates the optimal retrieval location derived via image processing. The system retrieves the quantity of the herb required to fill a designated number of packets, as shown in Figure 2(e). The drawer is then closed, as shown in Figure 2(f). The entire process (as shown in Supplementary Material available online at https://doi.org/10.1155/2017/9013508) is repeated until all of the herbs required for a given order have been retrieved.

In the above process, a database is used to establish the relationship between the size of the claw aperture and amount (weight) of the herb to be retrieved. This is meant to enhance retrieval accuracy and the overall robustness of the system when dealing with a wide range of herbs. To further enhance the efficiency of herb retrieval, we also calculate the discrepancy between the retrieved weight and target weight and set a threshold of acceptable error. The width of the claw can then be adjusted to compensate for errors in measuring the weight of the herbs.

### 2.3. Claw Width

**Figure 4:**
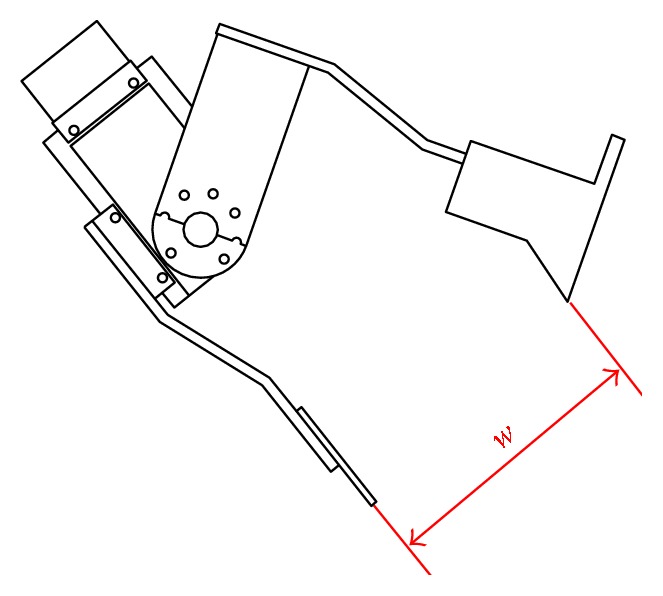
Schematic diagram showing the width of the claw *w*.

Herbs vary greatly in their size, shape, and unit weight. Even the same type of herb may differ considerably with regard to shape. To enable the adjustment of claw width during retrieval, we first derive the optimal relationship between the width of the claw and the weight of each type of herb and the results of which are stored in a database. As shown in Figure 4, the width of the claw is defined as the distance *w* indicated in the side view of the designed claw.

Three methods are used to estimate the relationship between the width of the claw and the weight of the herb: linear interpolation, curve fitting, and iterative refinement. The first method involves using a database to determine a weight range for a given herb and then applying linear interpolation to calculate the width of the claw needed to obtain the designated quantity of the herb. Suppose that the designated weight of the herb *g* and width of the claw *w* fall between two adjacent data points (*g*_*i*_, *w*_*i*_) and (*g*_*i*+1_, *w*_*i*+1_). Therefore, *g* and *w* fulfill the following equation:

(1) $$\begin{array}{c} g=g_{i}+\frac{w-w_{i}}{w_{i+1}-w_{i}}\times \left(g_{i+1}-g_{i}\right). \end{array}$$

The curve fitting method assumes that the herbs are evenly distributed within their drawers and fits a polynomial equation to obtain the relationship between the width of the claw and the weight of the herb. We performed curve fitting using least-squares regression (LSR) based on the retrieval results in the database and adopted a first-order polynomial as follows:

(2) $$\begin{array}{c} w=ag+b, \end{array}$$

where *a* and *b* are constants obtained via curve fitting. Both of the aforementioned methods employ the database established beforehand. Any major errors associated with the measurement data in the database affect the width of the claw and the actual retrieval results. Thus, we also developed an iterative refinement method to update estimates of how far the claw should be opened. Based on curve fitting, the proposed approach calculates the *R*-squared value and revises the retrieval equation repeatedly until reaching an acceptable *R*-squared value. The details are presented in Algorithm 1, where *y*_1_(*x*) and *y*_2_(*x*) denote the previous and current retrieval equations established using LSR and *R*_1_ and *R*_2_ are the corresponding *R*-squared values. If *R*_2_ is greater than *R*_1_, then the current retrieval equation *y*_2_(*x*) is deemed superior and is thus used to replace the previous retrieval equation *y*_1_(*x*); otherwise, the retrieval equation is not changed. When the current number of iterations *i* reaches the designated number of iterations *I*, then the experiment is terminated and the latest retrieval equation *y*(*x*) is used to estimate the width of the claw for herb retrieval.

**Figure 5:**
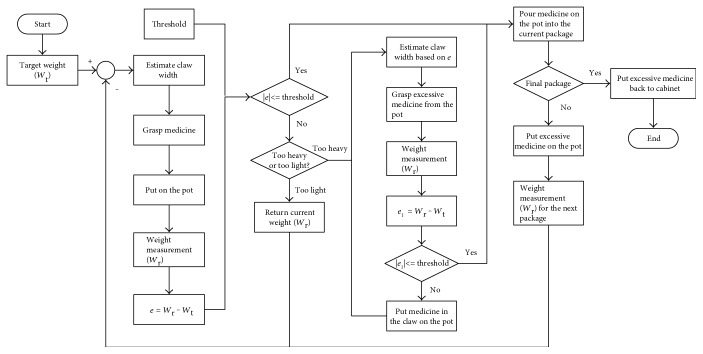
Proposed herb retrieval and dispensation process.

To minimize the unavoidable effects of weight error during the herb retrieval process, the system calculates the discrepancy between the target weight (*W*_t_) and the weight of the herbs on the weighing pan (*W*_r_). This is done after each retrieval to determine whether to continue or stop the retrieval of that particular herb. The procedure is detailed in Figure 5 and Supplementary Material. After calculating the current weight error (*e* = *W*_r_–*W*_t_), the system determines whether the absolute value of *e* is within the preset error threshold. If *W*_t_ has reached the designated weight, then the herbs on the weighing pan are automatically poured into bags on the turntable below, thereby ending the retrieval process. If the absolute value of *e* falls outside the error threshold, then there are two possible situations: too heavy and too light. The system begins by estimating the width of the claw based on *e*. If the retrieved sample is too light, then the system retrieves more of that material and sends back the current *W*_r_ value to continue retrieval of the same herb. If the retrieved sample is too heavy, then the claw is used to remove a quantity of the herb proportional to the excess weight. The weight error *e*_1_ (*e*_1_ = *W*_r_–*W*_t_) is calculated again, and the above process is repeated until the value of *e*_1_ falls within the preset error threshold. The material remaining on the weighing pan is loaded into bags. The system then determines whether it is currently engaged in filling the last packet. If so, then the herbs currently held in the claw are returned to the drawer. If not, then the claw places the herbs on the weighing pan, measures the current *W*_r_ value, and sends it back for the next retrieval. If the error (*e*) falls within the preset threshold, then the retrieval process is concluded.

### 2.4. Image Processing

**Figure 6:**
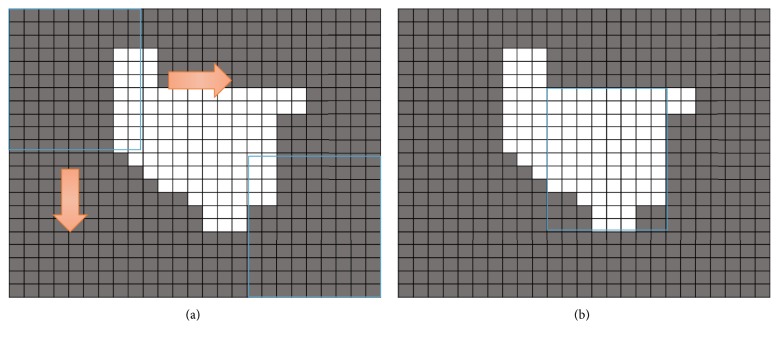
Herb density estimation. (a) Scanning method: from left to right and from top to bottom. (b) Final result with the blue frame as the ROI.

The location from which herbs can be retrieved changes every time a drawer is accessed. Thus, we employed two cameras to capture images and applied a series of preprocessing procedures to enable stereo vision monitoring. Preprocessing included blurring, color filtering, image binarization, and region of interest (ROI) estimation. The principle of parallax is used to create a stereo vision relationship for use in estimating the three-dimensional coordinates of the target retrieval location. We adopted the HSV format for color filtering: that is, hue (H), saturation (S), and lightness (V), with the range of the color channels serving as boundaries. Image pixels that fall within the preset HSV range are retained and the rest are filtered out. Image binarization involves setting the areas within the threshold as white and those outside the threshold as black. This accelerates the process by reducing computational complexity. Machine vision is used to identify areas in which the herbs are most densely distributed to which the claw is directed. The width of the claw is adjusted according to the designated weight of the herb. Therefore, we used the width of the claw to determine the corresponding ROI, which is used to calculate the pixel area of each region in the preprocessed image from left to right and from top to bottom, as shown in Figure 6(a) and Supplementary Material. The system selects the largest area as the target retrieval region.  Figure 6(b) presents an example of the scanning results, in which the white regions indicate areas that are retained after color filtering and the black regions are regions that have been filtered out. The blue frame indicates the ROI, and the range of which is determined by the width of the claw. During retrieval, the robotic arm regards the center of the ROI as the target retrieval location. After obtaining the coordinates of the center, the stereo vision relationship is used to convert the coordinates into world coordinates of the target location.

### 2.5. Stereopsis

**Figure 7:**
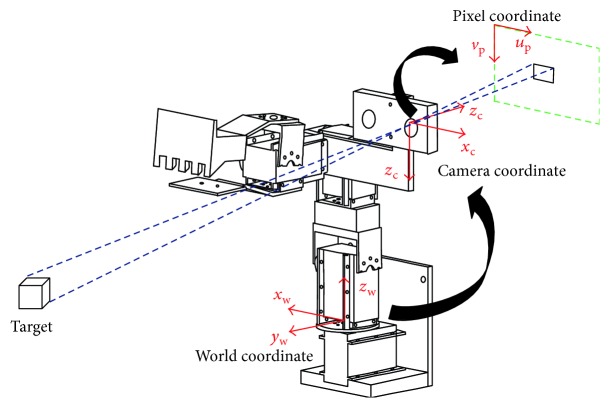
Coordinate system for stereopsis.

An appropriate coordinate system must first be established to calculate the correct location of the herb and obtain the spatial geometric relationships among the herbs, camera, and robotic arm in order to facilitate herb retrieval. We defined three coordinate systems for the various objects: world coordinates (*x*_w_, *y*_w_, and *z*_w_), camera coordinates (*x*_c_, *y*_c_, and *z*_c_), and pixel coordinates (*u*_p_ and *v*_p_). The conversion relationships are shown in Figure 7. For the pixel coordinates, the upper left corner of the images of herbs serves as the origin for use in describing the location of the target object on the image plane, whereas the coordinates for the center of the camera serve as the origin from which to describe the location of the target object. For the world coordinates, a point in the space is designated as the origin used to describe the absolute locations of various objects. The location of the target must be converted into world coordinates in order for the robotic arm to engage in tracking motions. In the conversion relationship above, the world coordinates are first converted into camera coordinates and then into pixel coordinates.

**Figure 8:**
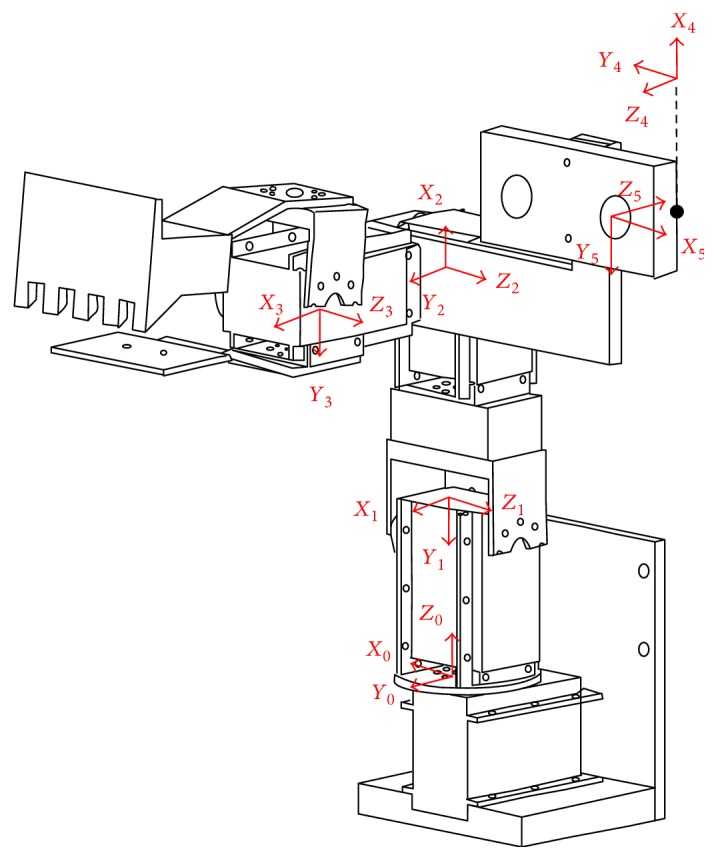
Camera coordinate system (frames 0–3: motor rotation axis; frame 4: left camera location; and frame 5: center of left camera).

The conversion relationship between the pixel coordinates and the world coordinates can be described as follows:

(3) $$\begin{array}{c} s\left[\begin{array}{c} u_{p}\\ v_{p}\\ 1 \end{array}\right]=\left[\begin{array}{cccc} f & 0 & 0 & u_{0}\\ 0 & f & 0 & v_{0}\\ 0 & 0 & 1 & 0 \end{array}\right]\left[\begin{array}{cc} R_{3\times 3} & T_{3\times 1}\\ 0_{1\times 3} & 1 \end{array}\right]\left[\begin{array}{c} x_{w}\\ y_{w}\\ z_{w}\\ 1 \end{array}\right], \end{array}$$

where *s* is a gain value; *u*_p_ and *v*_p_ are the coordinates of the target as described by the pixel coordinate system; and *x*_w_, *y*_w_, and *z*_w_ indicate the location of the target in the world coordinate system. *f* represents the focal length of the camera; *u*_0_ and *v*_0_ are the coordinates of the center of the camera, the parameters of which can be found via standard camera calibration [22]. **R**_3x3_ and **T**_3x1_ are the external parameters of the camera, respectively, denoting the rotation matrix and translation vector of the world coordinate with regard to the camera coordinates. In this study, the camera was installed on the third axis of the robotic arm, which means that the conversion relationship between the coordinate systems changes as the arm moves. Thus, a camera coordinate system must be defined to calculate the external parameters. Based on robotics theory, we constructed a D-H table (as shown in Table 1) to define the camera coordinate system shown in Figure 8, where *α*_*i*_ represents a constant twist angle; *a*_*i*_ denotes the vertical distance between the *z* axes of adjacent coordinate systems, generally equal to the link length; *d*_*i*_ is the joint offset; and *θ*_*i*_ represents the joint angle.

Based on this D-H table, we derived the conversion relationship using (4), where **E** is referred to as the external parameter of the camera in this study; *C*_1_ = cos*θ*_1_, *S*_1_ = sin*θ*_1_, *C*_23_ = cos(*θ*_2_ + *θ*_3_), *S*_23_ = sin(*θ*_2_ + *θ*_3_), and ^*i*−1^**A**_*i*_ is the matrix of conversion between the joints; *θ*_1_, *θ*_2_, and *θ*_3_ denote the amount of rotation in each joint in the robotic arm, indicating the current posture of the arm; *θ*_4_ is the angle between frame 3 and frame 4 in Figure 8; *θ*_5_ is the angle between frame 4 and frame 5, which does not change with the posture of the arm and is therefore constant. This conversion matrix makes it possible to convert the target location estimated in the camera coordinate system into coordinates in the coordinate system of the robotic arm. The conversion relationship is as shown in (5), where **P**_r_ and **P**_c_, respectively, represent the locations of the target as described by the coordinate system of the robotic arm and the camera coordinate system.

(4) E=A01A12A23A34A45=−S1−C1S23−C1C23−a5S1+d5C1C23+d4S1+a4C1S23+a3C1C23+a2C1C2C1−S1S23−S1C23−a5C1+d5S1C23+d4C1+a4S1S23+a3S1C23+a2S1C20−C23S23−d5S23+a4C23−a3S23−a2S2+d10001,

(5) $$\begin{array}{c} P_{r}={EP}_{c}. \end{array}$$

## 3. Results

**Figure 9:**
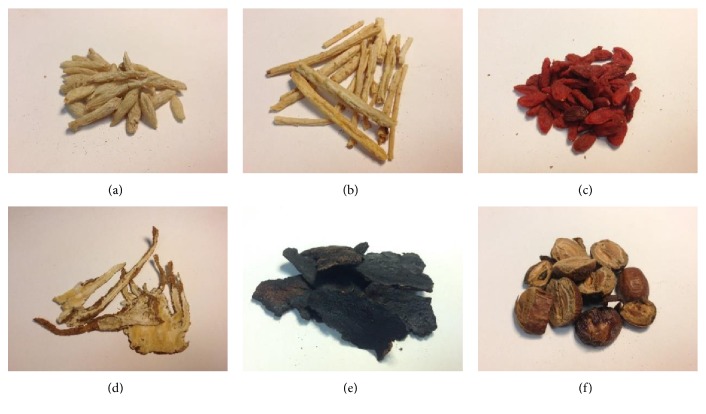
Photos showing the ingredients of Yi Guan Jian: (a) Dwarf Lilyturf Tuber, (b) Radix Glehniae, (c) Barbary Wolfberry, (d) Chinese Angelica, (e) Dried Rehmannia Root, and (f) Szechwan Chinaberry.

TCM practitioners prescribe traditional formulas (prescriptions with fixed compositions of particular ingredients) or make up their own formulas. In this experiment aimed at demonstrating the feasibility of the proposed ADD system, we selected the traditional formula referred to as Yi Guan Jian (一貫煎) [23]. As shown in Figure 9, this formula includes six herbs: Dwarf Lilyturf Tuber, Radix Glehniae, Barbary Wolfberry, Chinese Angelica, Dried Rehmannia Root, and Szechwan Chinaberry. As can be seen, these herbs vary widely in size, shape, and color.  Table 2 presents the formula of this prescription. The unit “mace” is commonly used for prescriptions in TCM.

**Figure 10:**
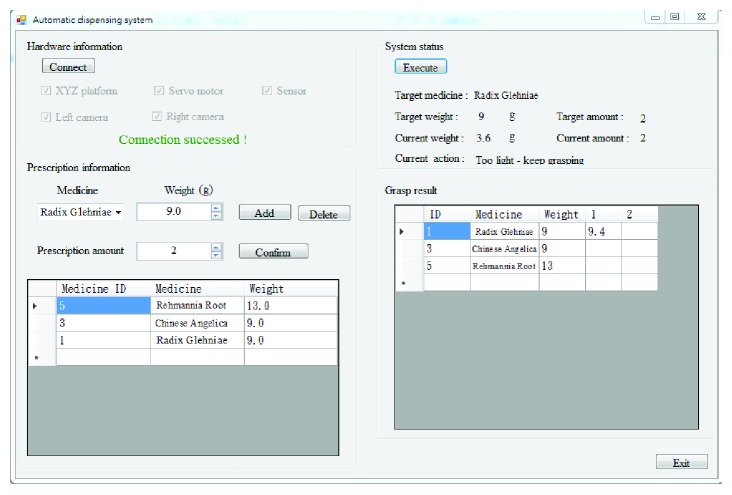
Human-machine interface for the automatic dispensing system.

As shown in Figure 10 and the Supplementary Material, we designed a user-friendly human-machine interface to enter the ingredients of prescriptions. The fields on the right display the target medicine, target weight, and target number of packets. The current weight and current amount represent the weight that the system has retrieved and the number of packets that have been processed. The action currently being performed by the system is also displayed in the current action field to enable monitoring by the user and facilitate debugging. As each packet is filled, the system lists each completed retrieval in the table in the lower right corner of the window, so that users can monitor the system's current progress. Below, we examine the database and the results obtained during the retrieval process. The database contains information pertaining to the relationship between the width of the claw and the weight of the herb for each type of herb. These values were derived using (1) linear interpolation, (2) LSR curve fitting, or (3) iterative refinement. We analyzed the retrieval results achieved using these methods for use in assessing the feasibility of the proposed ADD system.

### 3.1. Estimation of Grasp Volume

**Figure 11:**
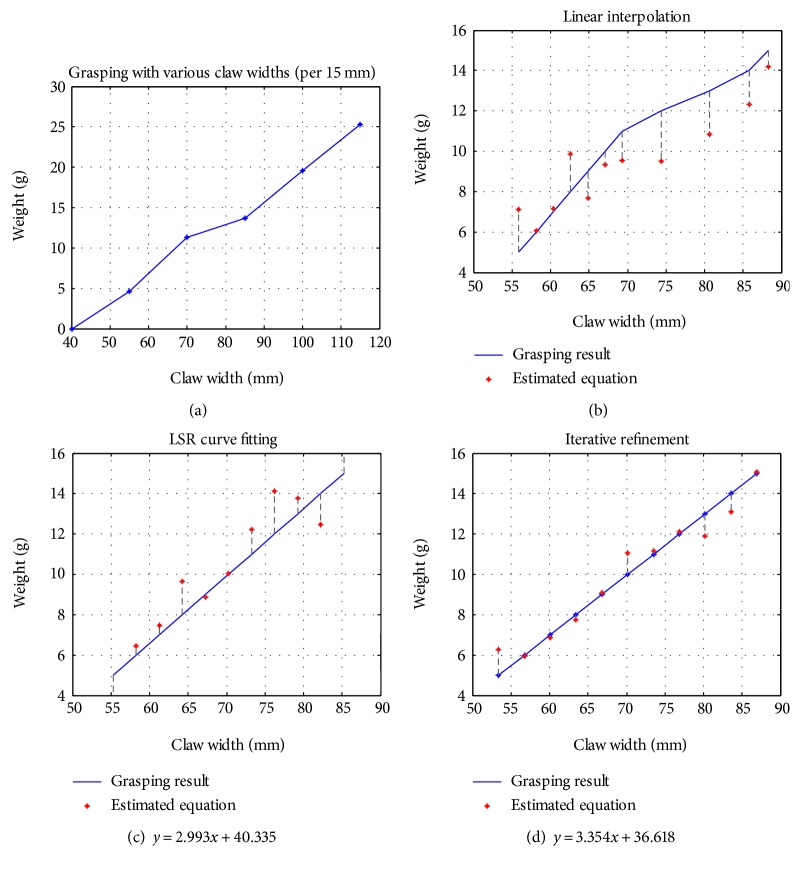
Estimation of grasping amount—Chinese Angelica. (a) Grasping with claws of various width (from 40 mm to 115 mm/per 15 mm). (b) Grasp estimates obtained using linear interpolation using the data in (a). (c) Grasp estimates obtained using LSR curve fitting with the data in (a). (d) Grasp estimates obtained based on LSR curve fitting after refinement (three iterations).

The purpose of this experiment was to investigate the accuracy of the system with regard to the volume of materials retrieved. We set the width of the claw at 40 mm, 55 mm, 70 mm, 85 mm, 100 mm, and 115 mm prior to performing retrieval motions. Using 1 g as the sampling point, the system preformed retrieval tests for quantities ranging from 5 g to 15 g in order to establish a herb retrieval database. We then used linear interpolation, LSR curve fitting, and iterative refinement to estimate the corresponding relationships between the width of the claw and the weight of the herb. Finally, we calculated the *R*-squared value of the retrieval results for comparison and analysis. For the sake of convenience, we use the retrieval results of Chinese Angelica in Figure 11 as an example.  Figure 11(a) presents the weight of the herbs corresponding to the width of the claws from 40 mm to 115 mm at intervals of 15 mm. Figures 11(b) and 11(c) show the relationships between the weight of the herb and the width of the claw derived using linear interpolation and LSR, based on the results in Figure 11(a).  Figure 11(d) presents the retrieval equation obtained after applying the LSR-based refinement method through three iterations (the algorithm mentioned in Section 2.3). In Figures 11(b), 11(c), and 11(d), the solid lines in the figures represent the claw estimation relationship, and the asterisks (∗) show the actual herb retrieval results derived using said relationships. As can be seen in Figure 11(b), most of the retrieval results calculated from the width of the claws using linear interpolation do not fall along the linear line that was estimated, and the errors present no fixed trend. A comparison of Figures 11(b) and 11(c) revealed that LSR curve fitting could be used to reduce retrieval errors; however, many of the weights would still be far from the ideal weights.  Figure 11(d) clearly shows that using the iterative refinement method to revise the retrieval equation estimated using LSR curve fitting can produce retrieval results that are very close to the values estimated using retrieval equation, that is, greatly reducing retrieval errors.

Table 3 shows the *R*-squared values derived from the retrieval results using the three methods. Clearly, curve fitting (method 2) achieved better *R*-squared values (all greater than 0.8) than did linear interpolation (method 1), regardless of the type of herb. The *R*-squared values from iterative refinement (method 3) were all greater than 0.9, nearing 1 for Dwarf Lilyturf Tuber. Due to the fact that the herbs were distributed unevenly, differences in the size and shape tend to result in different equations after curve fitting. Nonetheless, iterative refinement can improve the accuracy of retrieval equations obtained using curve fitting.

### 3.2. Analysis of System Performance

To examine the operating efficiency and accuracy of the proposed system, we went to an actual TCM pharmacy and had the pharmacist manually prepare five packets of Yi Guan Jian. We then used the width of the claws estimated using the three methods to analyze the performance of the ADD system. The acceptable error threshold was set to 2 g, that is, a retrieval weight within ±2 g of the target weight was considered acceptable.  Table 4 compares the manual retrieval results and the retrieval results of the proposed system. The average error, standard deviation of error, and max error were calculated after multiple measurements using an electronic scale (resolution 0.1 g).

Table 4 shows that errors greater than 2 g occurred in some cases. This was because the weight measurements were obtained using a strain gauge and the herbs were poured into the bag when the error values were less than the preset threshold. Following the retrieval process, the herbs in the bag were then weighed using a scale. The limited resolution of the strain gauge was the cause of the measurement error. Examination of the results revealed more pronounced retrieval errors with Chinese Angelica and Dried Rehmannia Root. This was because these two parts of herbs come in large pieces that are difficult for the excavator bucket-like claw to retrieve, particularly when the herb pieces are not arranged neatly. Our results clearly revealed uneven retrieval errors when manually dispensing Barbary Wolfberry and Dried Rehmannia Root. This is because pharmacists usually use a traditional Chinese scale (low resolution) to obtain rough values of scale readings and then rely on experience for subsequent dispensation processes. Therefore, the error results may vary every time that experiments are performed by different pharmacists or with different prescriptions. In terms of average error, setting an error threshold was shown to reduce retrieval errors. Nevertheless, a comparison of the results from the three methods still revealed some differences. With iterative refinement, the average error and standard deviation of error all remained at approximately 1 g, unlike those resulting from the other two methods (linear interpolation and curve fitting) and the manual method. Note that these errors could be further reduced by setting a smaller error threshold and/or using more advanced herb retrieval methods. Nevertheless, the proposed approach did not improve all of the retrieval results (such as those for Dried Rehmannia Root). This was because herb retrieval was set to proceed continuously in our experiment. The distribution of herbs in the drawer changed each time it was accessed, and sometimes the more elongated herbs were moved into positions that were difficult to access using the claw. This affected the depth of retrieval as well as the retrieval results for the next packet. Overall, iterative refinement was shown to reduce the average number of retrievals needed to complete a packet and thus the time required to complete an order. However, the total time needed was still significantly longer than manual retrieval. This may be due to the fact that we adopted an XYZ platform and a robotic arm to perform large-stroke motions. Insufficient structural rigidity resulted in shaking, which hindered the retrieval process. Furthermore, limitations in the moving speed meant that retrievals could not be performed quickly. Nevertheless, the experimental results demonstrate the accuracy and feasibility of the proposed approach.

## 4. Conclusions

This study proposed a robotic arm-based ADD system with machine vision to assist in the collection of ingredients for herbal decoctions. Experiments involving the collection of five packets of a prescription containing six types of herbs were conducted to demonstrate the feasibility of the proposed automated process and retrieval algorithm. Experimental results indicate that obtaining an appropriate retrieval equation for the width of the claw and retrieval weight using iterative refinement is an effective approach to enhancing retrieval accuracy and improving the operating efficiency of the system. Retrieval apparatus designed to simulate human fingers and structural enhancements to the system could further improve the operating speed and efficiency of the system. The inclusion of image-based machine learning technology to determine the optimal width of the claw and robotic arm decisions may also serve as solutions for the retrieval of herbs with complex shapes.

## Supplementary Material

Demo video for the proposed herbal dispensing system.

## Figures and Tables

**Figure 3:**
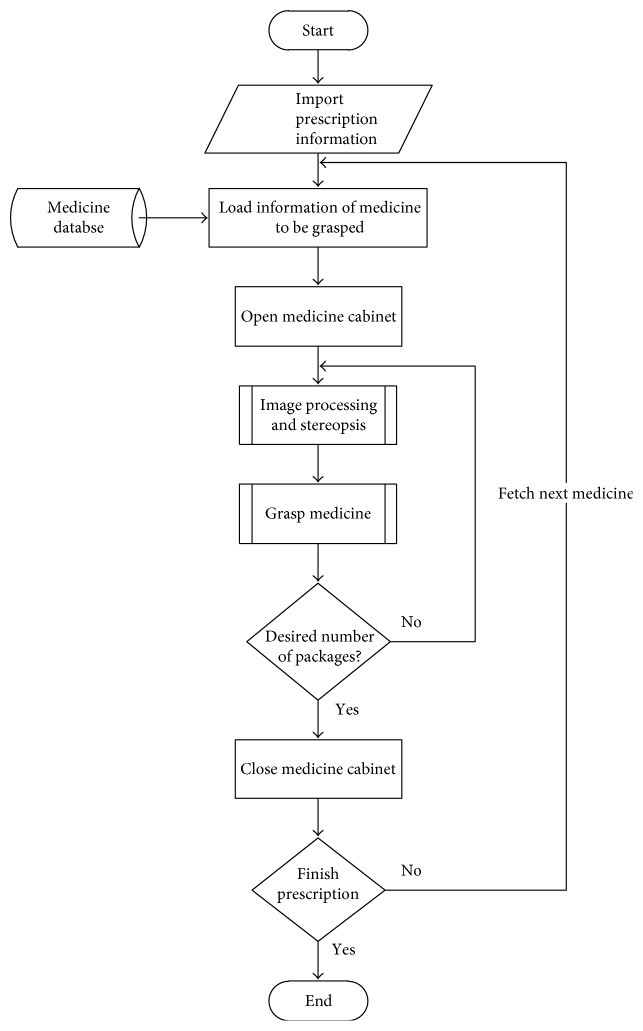
System flowchart.

**Algorithm 1:**
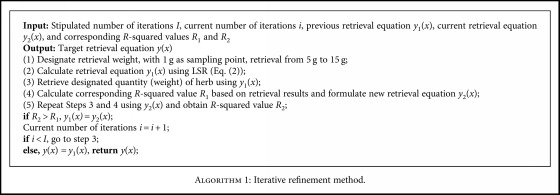
Iterative refinement method.

**Table 1:** D-H parameters for the camera coordinate system.

*i*	*α_i_*	*a_i_* (mm)	*d_i_* (mm)	*θ_i_*
1	90°	0	50.6	*θ*_1_
2	0°	107	0	*θ*_2_
3	0°	39	0	*θ*_3_
4	−90°	50	135	*θ*_4_(−90°)
5	180°	0	12	*θ*_5_(−90°)

**Table 2:** Ingredients of Yi Guan Jian.

Medicine	Quantity (mace)	Medicine	Quantity (mace)
Dwarf Lilyturf Tuber	3 mace	Chinese Angelica	3 mace
Radix Glehniae	3 mace	Dried rehmannia root	7 mace
Barbary Wolfberry	4 mace	Szechwan Chinaberry	1.5 mace

1 mace (錢): 3.125 g.

**Table 3:** Comparison of *R*-squared values using three methods (method 1: linear interpolation; method 2: LSR curve fitting; and method 3: iterative refinement).

Herbal medicine	Method 1	Method 2	Method 3
1 (Dwarf Lilyturf Tuber)	0.7737	0.8861	0.9617
2 (Radix Glehniae)	0.6281	0.8254	0.9382
3 (Barbary Wolfberry)	0.8886	0.8151	0.9172
4 (Chinese Angelica)	0.5452	0.8493	0.9464
5 (Dried Rehmannia Root)	0.8382	0.8481	0.9490
6 (Szechwan Chinaberry)	0.8316	0.9065	0.9417

**Table 4:** System performance analysis using five packets of herbs (medicine 1: Dwarf Lilyturf Tuber; medicine 2: Radix Glehniae; medicine 3: Barbary Wolfberry; medicine 4: Chinese Angelica; medicine 5: Dried Rehmannia Root; and medicine 6: Szechwan Chinaberry).

Herbal medicine	1	2	3	4	5	6
Quantity (g)	9	9	13	9	20	4.5
*Measured by pharmacist (five doses)*						
Average error (g)	0.91	0.91	0.4	0.93	0.44	0.98
Standard deviation of error (g)	1.02	1.45	0.65	1.24	0.57	1.41
Max error (g)	2.9	1.2	1.3	1.5	0.8	1.7
Executing time	9 min 27 s					

*Proposed dispensing system: linear interpolation method (five doses)*						
Average error (g)	0.92	1.12	1.2	1.16	1.08	1.06
Standard deviation of error (g)	1.02	1.28	1.35	1.37	1.32	1.23
Max error (g)	1.6	1.8	1.9	2.3	1.9	1.7
Number of average grasping (times)	1.6	1.6	1.6	1.4	2	1.2
Executing time	20 min 38 s					

*Proposed dispensing system: LSR curve fitting method (five doses)*						
Average error (g)	1.5	1.46	1.04	1	1.18	0.74
Standard deviation of error (g)	1.23	1.24	1.12	0.95	1.31	0.91
Max error (g)	2.3	2.3	1.4	2	2.4	1.6
Number of average grasping (times)	1.8	1.4	1.4	1.4	1.8	1.4
Executing time	20 min 11 s					

*Proposed dispensing system: iterative refinement method (five doses)*						
Average error (g)	0.64	0.9	0.94	0.72	1.14	0.72
Standard deviation of error (g)	0.81	1.01	1.04	0.91	0.93	0.82
Max error (g)	1.1	1.4	1.8	2.1	2.2	1.3
Number of average grasping (times)	1.2	1.2	1.4	1.2	1.6	1.2
Executing time	18 min 1 s					
